# Cancer

**Published:** 1881-01

**Authors:** 


### CANCER.

MANY persons undergo a good deal of mental suffering from the want of knowledge of one important fact in relation to this dreadful disease. It is this: When cancer commences, its march is steadily onward toward death, unless arrested; therefore any sore or tumor, however suspicious in appearance, which gets better, or which can be arrested by simple remedies, so that it improves or heals, is not cancer.

I do not say that such sores may not eventually become cancerous, where a tendency to the disease exists, and their liability to do so would probably be a little greater, as disease usually attacks the weaker points; but I do say that a sore that shows marked improvement from its worst condition should cause but little uneasiness or worry. It seems very difficult to determine with anything like certainty whether cancer is, or is not, an hereditary disease. Physicians are divided in opinion, just as they are in regard to the nature of the disease.

I have seen very many cases, and in all stages of development; and I am inclined to believe that the tendency to cancer is inherited—not that the disease exists as a blood poison, but that certain particles or nuclei exist by inheritance, which are liable under certain conditions to develop into cancer. I believe, more—over, that the prospect of eradicating the disease depends on its location, and upon the promptness with which the proper remedies are employed. If deep-seated they had better be left to themselves, and efforts made to keep up the general health. If superficial, it is possible to destroy them effectually, in many cases, because they exist at first as local affections, the blood remaining for sometime uncontaminated. There resides in New York to-day, a physician, who twenty years ago lost the entire under lip by cancer. He says he arrested the disease by applying tannin to the sore every day and, on the day following, standing in front of a looking glass, he could see small black spots in the sore; these were the upper extremities of the roots of the tumor, and, seizing them with a small pair of forceps, he would draw them out. He extracted very many in this way; in fact, all that existed. Many of these he had preserved between plates of glass and showed them to me. They were from one to two inches long, and resembled the rootlets of a plant. In HALL'S JOURNAL OF HEALTH for December, 1877, I gave the history of a case of undoubted cancer, that was cured by electricity. The patient was a most intimate friend, and my anxiety was great, for not a symptom was wanting on which to rest a doubt as to the true nature of the disease ; it had extended rapidly to the ulcerative period, and seemed to me an almost hopeless case. My associate physician took a different view of the case, and it proved to be the correct one.

His theory was that cancer is at first a local affection, an independent and foreign body, and that the system does not become contaminated until ulceration has extended far enough for the matter to be absorbed into the system ; that strong currents of electricity passed through the tumor would kill it, and that in a few days it would shrivel up and become detached from its seat, leaving the wound in a healthy condition. Such proved to be the case. In five weeks from the first application of electricity no sign was to be seen that any sore had ever existed, and the patient has been in the enjoyment of his usual good health ever since.

